# Preparing researchers for patient and public involvement in scientific research: Development of a hands‐on learning approach through action research

**DOI:** 10.1111/hex.12671

**Published:** 2018-02-08

**Authors:** Maarten de Wit, Anna Beurskens, Barbara Piškur, Esther Stoffers, Albine Moser

**Affiliations:** ^1^ Stichting Tools patient empowerment Bussum the Netherlands; ^2^ Research Centre for Autonomy and Participation for People with Chronic Illnesses Zuyd University of Applied Sciences Heerlen the Netherlands; ^3^ Department of Family Medicine, CAPHRI School for Public health and Primary Care Maastricht University Maastricht the Netherlands; ^4^ Huis voor de Zorg Sittard the Netherlands

**Keywords:** coaching programme, community of practice, health researchers, patient and public involvement, patient participation

## Abstract

**Background:**

Acquiring the theoretical and practical knowhow of conducting patient and public involvement (PPI) in research is not part of the traditional curriculum of researchers. Zuyd University of Applied Sciences and Huis voor de Zorg, a regional umbrella patient organization, therefore started a 1.5‐year coaching programme.

**Objective:**

To establish a community of practice by developing a PPI coaching programme for senior and junior health services researchers of Zuyd University. The context consisted of research projects conducted by the participants.

**Methods:**

A participatory action research methodology. Data were collected from reports of thematic group meetings and individual sessions with participants, field notes and regular reflection meetings with the project team. Data were analysed by reflexive deliberation.

**Findings:**

The programme comprised a kick‐off meeting (52 attendees), followed by 7 group meetings with 11 junior and 9 senior researchers. The project team constructed a serious game based on the concept of the participation ladder. Questions and concerns differed for junior and senior researchers, and separate tailored meetings were organized for both groups. Between group meetings, participants received individual assignments. Group meetings were accompanied by individual coaching sessions to provide tailor‐made feedback. The programme concluded with a combined meeting with all stakeholders.

**Conclusion:**

Building a community of PPI practice through action research facilitates the development of a coaching programme that fosters social learning, empowerment and the development of a shared identity concerning PPI. The role and responsibilities of senior researchers should be distinguished from those of junior researchers.

## 1 INTRODUCTION

Patient and public involvement (PPI)^1^ is becoming a common feature in health research, as is the provision of appropriate support and education of patients who participate in research.^1^, ^2^ Publications on PPI often report on the efforts of research institutes to prepare patients for their new role as advisor, reviewer or collaborative partner, by developing lay summaries, glossaries, introduction sessions and training courses.^3^, ^4^ In contrast to the attention given to patients, there is hardly any literature available about supporting and guiding researchers in their new role as facilitator and supporter of PPI in their research projects. PPI is rarely part of the basic research curriculum of PhD candidates, and they face several challenges when they want to start engaging patients.^3^, ^5^, ^6^ They lack knowledge on concepts of PPI and ways of applying them in practice.^7^, ^8^, ^9^, ^10^ Not knowing the benefits and pitfalls of different options and their impact on the research outcomes is one of the reasons for not using the most appropriate PPI methods.^11^ Other reasons for limited use of PPI by researchers are reluctance to share control over the research agenda, resistance to change, time pressure and tokenism that is engaging patients only to meet funding requirements. Reasons from the perspective of patients are doubts about the value of personal experiences in research, scientific jargon, questions of representativeness, personal health conditions and not knowing what is expected from them.^6^, ^7^, ^8^, ^9^, ^12^, ^13^, ^14^

To overcome these challenges, it is not only patients who need education, but researchers also require practical tools, recommendations and structured training to show the benefits of PPI and provide guidance on ways to customize PPI methods for specific research projects.^7^, ^15^ Examples of educational programmes and materials have only recently become available. The INVOLVE website^2^ offers information about PPI modules for students attending an MSc Clinical Research programme and researchers at King's College, London University, as well as a virtual workshop for junior researchers, a one‐day training course for senior researchers and a training course for Research User Group Support Workers at the Arthritis Research UK Primary Care Centre in Keele.^3^

In Australia, the Consumer and Community Involvement Programme developed and evaluated a half‐day workshop for researchers working in public health and medical research.^16^ This workshop aimed at “increasing awareness of consumer and community involvement; changing attitudes to future implementation of involvement activities and influencing behavior in the methods of involvement used.” The workshop covered fundamentals of consumer and community involvement: why it is important, benefits of and barriers to involvement, ethical considerations and methods of implementation, in particular the need to find “suitable people.” After attending the workshop, the number of participants who found PPI very relevant had doubled, and almost all confirmed that the workshop had increased their understanding of PPI. Qualitative research among UK trialists and patient representatives involved in trial steering committees found less enthusiasm for the need to train researchers, in particular from the perspective of patient research partners, who felt that researchers “already possessed the skills needed.” Although some researchers questioned the evidence base for training researchers, they did identify a need for guidance on how and when to involve patient representatives and in particular the challenge of finding “suitable people.”^17^ The study concluded by suggesting that alternative types of education, such as coaching, were suitable to learning about PPI, maybe even together with patient representatives, to learn from each other. It also encouraged further efforts to conceptualize, design and deliver PPI training to researchers, to convince them of its relevance and support uptake.

Some studies and systematic reviews provide a first overview of PPI concepts that addresses the challenges to researchers^2^, ^18^, ^19^ and suggest conditions that are relevant to include in a coaching programme for researchers. These conditions are involvement of patients from an early stage; supporting patients to work in pairs; formulating common goals; clarifying mutual expectations and responsibilities; encouraging dialogue and co‐learning; and finally regular evaluation and feedback on processes and outcomes of PPI.^7^, ^20^, ^21^, ^22^ Many studies on PPI emphasize the added value of the participation ladder model^23^ in distinguishing levels of involvement in terms of contributions and opportunities to influence the research. The FIRST framework for PPI (*F*acilitate, *I*dentify, *R*espect, *S*upport, *T*raining) provides arguments to involve both senior and junior researchers.^5^, ^7^

It is clear that researchers need evidence to justify their PPI efforts and guidance on ways to apply the above‐mentioned concepts in daily practice.^7^ The literature also suggests that sustainability should be ensured by creating a “soft” infrastructure, including a culture of participation and a solid set of policies, rules and procedures.^3^, ^5^ This means that educating researchers should be combined with the establishment of a community of practice that provides active and long‐term support and facilitation of senior and junior researchers.^24^ It may even require a new approach to education and coaching to really enhance the implementation of meaningful PPI and to reduce the risks of tokenism. What is needed is a programme or coaching approach where researchers can simultaneously learn about PPI, debate personal values and beliefs, share practices and experiences on a regular basis and develop a shared identity around PPI. They also need an approach that integrates strategies at the level of individual researchers with those of the research institute. This is why in 2013, senior and junior researchers of the Research Centre on Autonomy and Participation of People with a Long‐term Condition at Zuyd University of Applied Sciences The Netherlands developed a systematic approach to the implementation of PPI in research projects.^25^

The objective of this article was to describe and reflect on the development of a hands‐on learning approach through a process of shared learning, debate and the exchange of experiences, supported by the provision of relevant evidence. This approach focused on encouraging researchers to consider and explore PPI methods, and to develop a critical though supportive attitude towards PPI and skills to implement PPI in practice. The ultimate goal was to establish a community of PPI practice for PPI through action research. We believe that it is particularly the managing directors and senior or principal researchers who are responsible for supporting and mentoring junior researchers or those novel to PPI in research who could benefit from our findings.

## 2 METHODS

### 2.1 Project team

The project team comprised 2 senior researchers and 2 patient experts: the leading investigator at Zuyd University of Applied Sciences, with a background in nursing (AM); a specialized public involvement staff member of Huis voor de Zorg, an umbrella organization representing patient organizations in the province of Limburg (ES); an external PPI expert with a PhD in participatory research and personal experience of a long‐term condition (MW), and a senior researcher who had gained extensive experience of patient collaboration in her PhD project at Zuyd University (BP). The team had regular face‐to‐face and Skype meetings to prepare for and reflect on the development of the PPI coaching programme and the community of PPI practice. The level of PPI pursued in this research team was that of partnership in every research phase: partnership in the sense of doing together and deciding together. The roles of the members were alternately those of initiator, educator, facilitator, coach and finally co‐author.

### 2.2 Action research and reflexivity

We used a participatory action research^26^ methodology to facilitate the combination of developing a coaching programme for and with researchers and, at the same time, establishing a community of PPI practice. We applied the action research cycle of observe, reflect, plan and act.^27^

Reflexivity was the theoretical orientation that guided us in the coaching activities as well as the analysis of the data collected and to a large extent also the participants in the meetings with their peers and mentors. We view reflectivity as a continuous dialogue and critical self‐evaluation of our positionality, with the explicit recognition that this position may affect the learning process and outcome.^28^ The coaching, data collection and analysis were interdependent and partly based on the project team members’ interpersonal, social and institutional context.^29^

We reflected on the development of a community of PPI practice, guided by Wenger's definition of a community of practice: a group of people “who share a concern or a passion for something they do and learn how to do it better as they interact regularly.”^30^ In our project, a community of PPI practice was defined as a group of researchers engaged in a long‐term process of collectively learning how to conduct and improve PPI in health research. Collective learning took place through the development of a PPI coaching programme, comprising a variety of methods such as teaching, coaching, serious gaming and sharing. The purpose of this community of PPI practice was to facilitate a bottom‐up process of gaining self‐confidence and knowledge about PPI in research. This was done by encouraging researchers to exchange and discuss values and attitudes regarding PPI and to learn from each other how to do better,^31^ but also by reflecting on the progress of the collaboration between stakeholders and on the changes required to the original approach and design of the coaching programme. In this way, reflexivity was consistently used during the individual and group meetings with participants, as well as during project team meetings.

We defined coaching as an activity that aims to support and empower researchers in developing their knowledge, values, attitude and skills regarding PPI in research.^32^ As mentioned above, the development of the coaching programme was based on an emergent design process of co‐creation in which a variety of learning methods were applied, such as plenary presentations, individual assignments, coaching sessions, individual conversations and coaching, and provision of background information. The development of the coaching programme took place simultaneously with the establishment of the community of PPI practice.

### 2.3 Setting

The setting was the Research Centre on Autonomy and Participation for People with Chronic Illnesses of the Faculty of Health of Zuyd University of Applied Sciences. This is a fast‐growing relatively young research group, specializing in applied research, with a clear ambition to conduct socially responsive research with an emphasis on PPI. It therefore focuses on supporting target groups (eg patients), improving professional practice and facilitating implementation processes together with health‐care providers. Researchers have various backgrounds, including health scientists, epidemiologists, speech therapists, occupational therapists, physical therapists and nurses. The purpose of the present project was to motivate, instruct and guide researchers in facilitating or conducting PPI in research, often in the context of ongoing PhD projects.

### 2.4 Participants

Participants in the coaching programme were 11 senior and 9 junior researchers who were affiliated with the above research centre. Senior researchers were principal investigators with a PhD degree, who apply for research funding and supervise projects. Junior researchers had less than 3‐year research experience and carried out research activities, often working towards a PhD degree. In total, 74 persons were involved at different time points of the project, including over 50 participants at the kick‐off meeting.

### 2.5 Process of data collection

From April 2014 to October 2015, we used an approach involving participative research with an emergent data design. A multimethod process of data collection was used, which emphasized iteration between planning, acting, observing and reflecting, as recommended in action research. Data were collected by means of minutes and reports of formal presentations (n = 5), all interactive coaching sessions (n = 6) and regular project team meetings (n = 11). Field notes were taken at the individual meetings with participants (n = 14) and of several informal communications (n = 19) as well as numerous e‐mail communications. The coaching sessions (n = 6) were recorded. All data collections centred on information needs, reflection on lessons learned and the challenges faced when engaging patients in a meaningful manner. At first, the data collection concentrated on the process of initiating PPI, including aspects such as which patients should participate in what research phase, identifying the “right” patient, expectations and roles of patients, patient role description and recruitment and representativeness of patients. During the course of the coaching programme, the junior researchers gradually acquired a set of best practices for PPI in research, and data collection then came to centre on practical issues such as budgeting, barriers and facilitators at individual, team and project level and also ethical issues such as the danger of overburdening patients. Towards the end, data collection centred on the sustainability of PPI in single projects but also in the entire research group and on conditions for successful PPI. The project team reflected on the process and outcomes of previous sessions and prepared the objectives and format of the next group session and the individual coaching. Written informed consent was obtained of all participants to use the information, data and reports collected during the coaching project.

### 2.6 Analysis

Data collection and analysis took place simultaneously. Our analysis was guided by a reflective process where data were analysed inductively.^33^ We read and re‐read reports, minutes, e‐mail correspondence and field notes and reflected upon the data. First, we identified and selected relevant text fragments and assigned descriptive codes. Next, we identified meaningful clusters within each data source and across the various data sources. We then compared, contrasted and reflected upon these clusters and pre‐categories emerged, after which main categories and subcategories were formulated. This was a process involving the project team going back and forth between the data, coding and producing reflective accounts. We used the (preliminary) findings to guide the coaching programme and inform subsequent activities. Finally, we checked whether the categories were stable and provided sufficient depth. We applied multiple strategies to ensure trustworthiness (Table 1).

**Table 1:** Strategies to ensure trustworthiness of the study

Method triangulation: we used multiple methods of data collection: minutes and reports, field notes, e‐mail exchanges and video and audio records.
Researcher triangulation: multiple researchers reflected on the methods, analysis process and findings. We reviewed and discussed scientific and organizational aspects of the study within the research team.
Data triangulation: we used multiple data sources during the study to verify the results, such as junior and senior researchers and patients, various written documents and reflective and analytical notes.
Thick description: we gave a rich description of the study context and process to enable readers to judge whether the findings are transferable to other care and research contexts.

## 3 FINDINGS

### 3.1 Coaching programme

The coaching programme “Preparing researchers for user involvement” included a range of components to guide the participants in implementing PPI in their research projects. Table 2 provides an overview of the structure, aims and content of the programme. The main components of the programme were 6 coaching sessions with pre‐session assignments, written feedback and several individual sessions.

**Table 2:** Coaching programme: Preparing researchers for patient and public involvement (PPI)

Meetings	Content	Seniors	Juniors	Patient research partners
Masterclass	General introduction to concepts of PPI by an expert to set the scene	N = 74 in total		
Individual assignment	Researchers are asked to prepare for session 1 by preparing a brief presentation of their research project and the role of PPI.		N = 6	
Coaching session 1	Discussion on the added value of PPI in research and exploring the participants’ expectations of the coaching programme.	N = 8	N = 6	
	Aims: (i) Discussing mutual expectations regarding the coaching programme; (ii) obtaining insight into one's own expectations regarding PPI and those of patient representatives; (iii) clarifying the different roles of senior and junior researchers; (iv) creating awareness about and sensitizing to PPI in research.			
Individual assignment	Researchers are asked to prepare for session 2 a profile of the patients to be involved in their research project.		N = 5	
Coaching session 2	Discussion about the ideal patient representative: what are appropriate criteria for recruitment and selection and how to formulate a role description (profile).	N = 7	N = 5	
	Aims: (i) learning how to write a clear and detailed patient partner profile, appropriate for one's own research project; (ii) exploring recruitment strategies.			
Individual assignment	Junior researchers are asked to finalize the role description (profile) and recruitment method for patient(s).		N = 7	
Individual coaching	If requested by the junior researchers		N = 7 (2 juniors twice)	
Coaching session 3	Discussion of the ideal PPI using the participation game.		N = 5	
	Aim: learning how to formulate the “ideal” design of PPI to fit the requirements of one's own research.			
Individual assignment	Junior researchers were asked to start implementing the PPI in research projects		N = 11 (all but one junior twice, one‐three times)	
Coaching session 4	Progress of implementation: reflecting on implementation success. What are facilitating and constraining factors?		N = 4	
	Aim: (i) exchanging experiences of implementing PPI approaches; (ii) sharing “best practices”; (iii) discussing constraining and facilitating strategies.			
Individual coaching	If requested by the junior researchers.		N = 7 (2 juniors twice)	
Coaching session 5	Sustainability of the infrastructure for PPI across the institute.	N = 6		N = 1 (patient representative Huis voor de Zorg)
	Aim: (i) exchanging experiences of implementing PPI approaches; (ii) discussing the infrastructure required for sustainable implementation.			
Coaching session 6	Closing session: discussions among all stakeholders and final evaluation of the coaching programme.	N = 6	N = 5	N = 3
	Aim: discussing and reflecting on conditions for PPI			

### 3.2 Preparing researchers for user involvement

In April 2014, a kick‐off meeting was organized for a large and diverse audience including third parties like research assistants, lecturers involved in research, management staff and researchers from other research programmes. A masterclass was organized which functioned as the kick‐off meeting for the coaching programme. During this masterclass, the potential added value of PPI was emphasized, followed by a discussion of best practice for patient involvement in the development of a patient‐reported outcome measure. This best practice example elaborated on the different levels of PPI in different phases, based on the ladder of participation. The masterclass was videotaped and made available as an open resource.^34^

The content of the coaching sessions that followed focused on the researchers’ expectations and perceived value of PPI. A simplified Dutch version of the participation ladder^6^ played a pivotal role in introducing and guiding the researchers in initiating, designing and conducting PPI.

The first group session explored the PPI experiences of the participants, discussing successes as well as failures and the individual participants’ questions.

*[name senior researcher] would like to learn more about which level of patient participation fits which research questions? And, most patient representatives are too professional and have little contact with the group they represent. How can I deal with this?* [minutes of session]

During the second coaching session, participants discussed the ideal design of PPI and implications for the recruitment and selection of patient representatives. Based on their own study, researchers were invited to write a role description for a patient representative, including a set of required competences. Participants learned that in some studies, the patient representatives involved do not have to be the same in all stages. After this session, it became apparent that the interests and queries of the senior researchers deviated from those of the junior researchers. The seniors not only indicated they did not have enough time to attend all coaching sessions, they also realized that their responsibilities were to advise, support and supervise juniors in doing PPI, rather than maintaining direct contact with patients. The group agreed to continue the coaching programme in 2 separate streams, one for senior researchers, focusing on the establishment of supportive conditions for junior researchers and one for junior researchers, focusing on the practicalities of engaging patients in research projects. In Box 1, we reflect on the implications of this decision related to power issues.

One junior participant was reluctant to continue the programme. However, after explaining that the programme would not prescribe what should be done, this researcher stayed and became more interested.

Box 1 **Team reflection on power dynamics**

1 During one of our research team meetings, we discussed the potential implications of the separation of the junior and senior groups for the relationships between juniors and seniors. Was this a consequence of existing power imbalances or a logical result of becoming engaged in a community of PPI practice?

Power dynamics play an important role in the education of students and research fellows. Hierarchical relationships are a challenging factor in the context of a community of practice where learning is based on principles of equality. When senior participants noticed that their needs and roles differed from those of the juniors, the research team had to reflect on the implications of this unexpected feedback. Three considerations were important. First, equality in the research team between the 2 researchers and the 2 patient representatives was essential principle before the start of the project. These relationships in the research team were intended to reflect the desired relationships in the coaching programme. Second, the wish to separate the coaching programme of the seniors from that of the juniors was not motivated by intentions to maintain the status quo, but, on the contrary, to address the specific challenges and needs that were shared by the juniors. They became aware that in the new situations their role, behavior and attitude needed to change. Just like the juniors, who indicated that they valued the peer‐to‐peer feedback in the programme, the seniors recognized the benefits of a separate and safe environment to discuss their new facilitative role. Finally, juniors shared their experiences with their own mentors, who did not participate in the coaching programme. Our project team identified 2 different patterns of resistance, one on the part of the mentors and one among the juniors. In the first case, it was the juniors who experienced difficulties justifying their PPI efforts in the eyes of mentors who were not supportive of the concept of PPI. In the second case, it was juniors who reported difficulties with their mentors who demanded too much PPI.

### 3.3 Junior researchers

**Figure 1:**
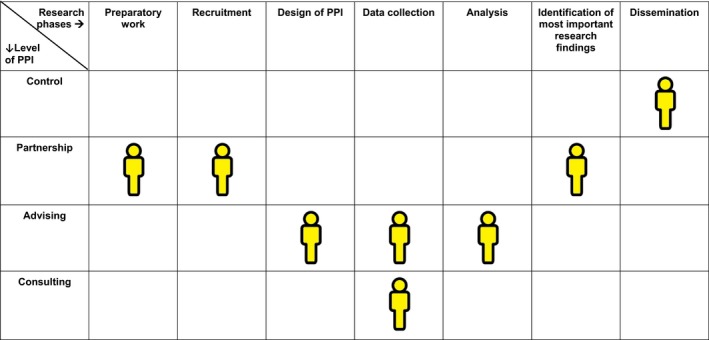
Participation matrix. The participation matrix gives an overview of patient and public involvement (PPI) within research projects based on the “participation game.” For example in data collection, PPI on advising means the advice of patient partners in research and consulting means PPI as participants in an interview

During the third coaching session for junior researchers, we introduced the participation game. This is a serious game that provides an interactive and safe playing field for dialogue (see Box 2). The participation game aims to support researchers to identify the appropriate roles and tasks of patients throughout the research process. It is based on the model of the participation ladder^23^ and has proved to be a helpful concept in the context of our programme to convey opportunities and options regarding PPI (see Figure 1). We used the version developed by Haarsma and colleagues,^6^ which builds on the original concept of Arnstein,^23^ but without its normative and hierarchical connotations. We replaced the purpose of “maximal participation” with “meaningful participation,” defined as the level and form of participation that is feasible and acceptable to both researchers and patients within a given context. These contextual factors were then explored and divided into personal factors such as preferences (eg avoiding overburdening) or capacities (eg previous PPI experience or health condition), and structural factors such as resources (eg time, funding, existing deadlines). By incorporating these contextual factors in the way we used the participation ladder, we made PPI more tangible for the participants. Since the participation game taps into the explicit problem‐solving capacities of the participants and their colleagues, it enabled them to formulate facilitating as well as constraining factors that were specific for their individual research project.

Box 2 **Serious gaming—the participation game**

1 A matrix is laid out on the floor. The rows represent the level of PPI: information‐consultation‐advising‐partnership and control. The columns represent the phases of research. These phases might be subdivided into specific research activities. The facilitator explains the rules and addresses the group dynamics. Researchers sit around the participation matrix.

The researcher whose turn it is describes the research projects, then in which research phase or activity they want to use PPI and why, and what participation mechanism they want to apply. He/she takes a pawn and places it in the square he/she perceives as appropriate. During this procedure, the other researchers, who sit around the matrix, should not ask questions or discuss the explanations of the researcher whose turn it is. This encourages them to listen. When the researcher whose turn it is has finished, the other participants sitting around the matrix are allowed to ‘enter’ the game, ask further questions, discuss or give advice. The researcher whose turn it is might then shift the pawns again. At the end, a photograph is taken and the researcher whose turn it was has literally pictured the design of PPI, which he/she will develop further. This procedure is followed until each researcher has had their turn (see Pictures 1 and 2 and Figure 1).

**Picture 1:**
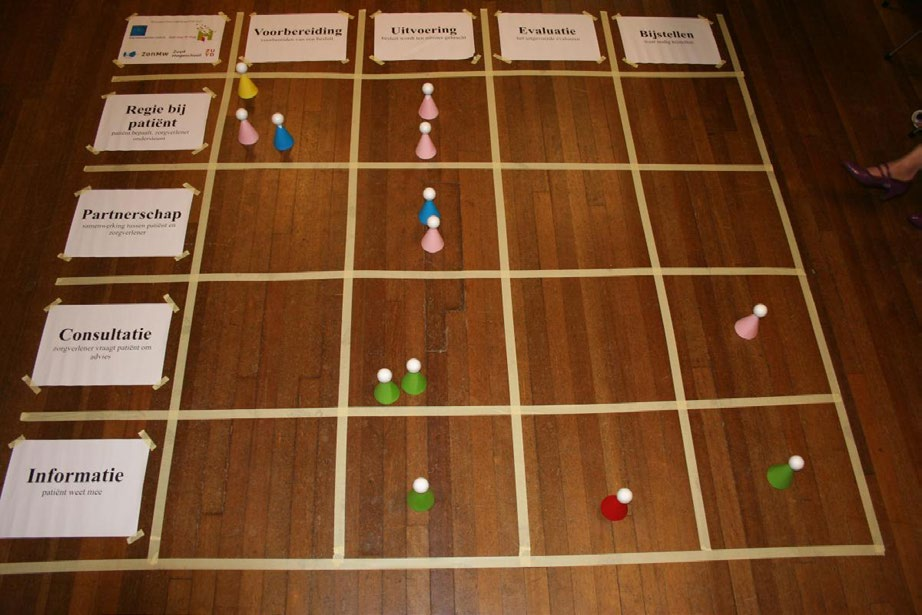
Picture 1Pawns reflecting the desired level of patient involvement

**Picture 2:**
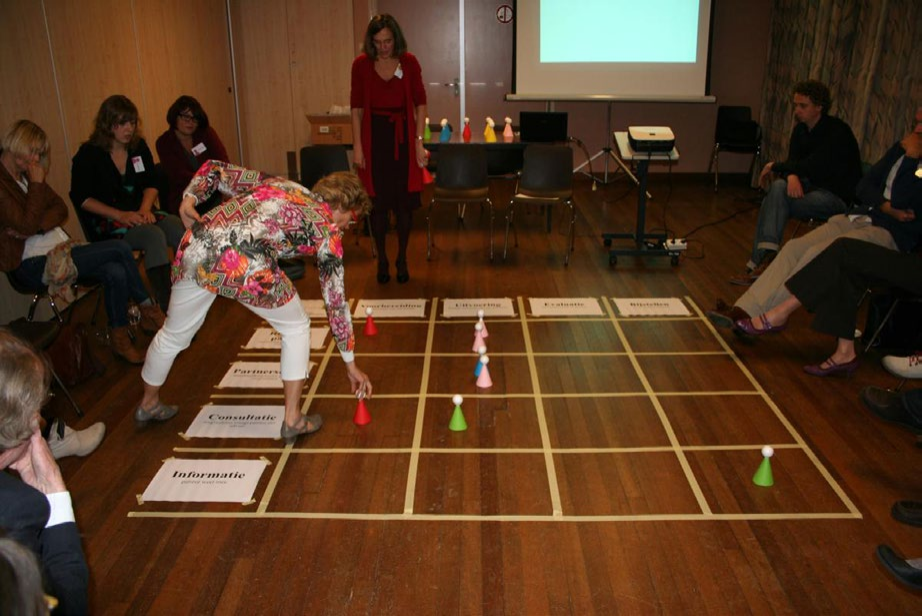
Picture 2Participants playing the participation game

*Patients and patient representatives are out there. For me it's unclear if there are patient organizations that I can contact. We have discussed neither in our research group nor in my project team who are our preferred patient groups or patient organizations. I don't know if colleagues have a network and if I could make use of it*. [session recording]

We particularly collected structural challenges related to the research centre and forwarded these to the sessions of the seniors (Table 3).

**Table 3:** Structural challenges identified by the junior researcher participants

1. Unclear which organizations to contact for recruiting patient representatives: Does the research centre have preferred organizations to consult or to work with? Does the institute keep records of existing working relations or contact persons for patient organizations?
2. Patient and public involvement (PPI) insufficiently specified in research proposals: How much PPI can be done without missing important deadlines?
3. Uncertainty about the priority of PPI in the views of the research centre. How much time am I allowed to spend on PPI? Will I be supported by my superiors when input of patient representatives leads to changes in the design, conduct or outcomes of my study?
4. Lack of guidance on how to budget for meaningful PPI
5. Absence of a policy for remunerating patient representatives in research projects. For example: Is it possible to pay a symposium fee for a patient representative? Is there a budget for child care for a patient representative? Is it allowed to pay patient representatives for their involvement? And if so, what is a reasonable compensation?
6. Lack of guidance on recognizing the contributions of patient representatives, not only individually, but also at the level of the research centre.
7. Do regulations exist for acknowledgement or co‐authorship of documents, brochures and scientific manuscripts?

Junior researchers presented a design for participation in their own research on a matrix drawn on the floor. By walking around and moving pawns across the matrix, they discussed expectations and feasibility of different forms of participation. The discussions and reflections stimulated them to consider more equal and sustainable forms of partnership. Some were eager to experiment with extensive forms of participation. Their ambition sometimes needed to be toned down to avoid disappointments due to unachievable aims. Others were more reluctant and did not want to go beyond the level of “asking for advice.” The practical use of the participation ladder was an eye‐opener, and the format of the game was experienced as helpful and enjoyable:

*(…) the participation game was very helpful. The game enables you as a junior to take part in patient participation as well as to observe it’ Inside because I design patient participation for my own research and literally move across the matrix, outside because I see how my fellow juniors design patient participation for their own projects. Then we discuss and give feedback to each other*. [session recording]

Following the principles of reflective learning, the junior researchers discussed successes and lessons learned regarding the PPI implementation process. Brief individual conversations made clear that the junior researchers felt they had received sufficient tools to successfully start working with different forms of PPI. They mentioned the role descriptions, participation ladder, participation matrix, written feedback on assignments and the individual coaching.

Common among all junior researchers were practical issues, mostly related to “how‐to‐do” questions and perceived barriers. One topic that cropped up frequently was the difficulty of recruiting “the right patients,” which sparked discussions on what constitutes the “right patient.” Another topic related to the budget for PPI: What are the financial implications of PPI and what are realistic and feasible opportunities to compensate patients? This issue was forwarded to the seniors.

Although junior researchers were willing to initiate some extra activities to implement PPI in their research, some reported that seniors considered PPI as nice‐to‐do but not necessary‐to‐do:

*In my research team patient participation is perceived as just a “nice” activity. I will put patient participation on the agenda of every project team meeting*. [session recording]

The fourth coaching session with junior researchers centred on PPI implementation themes, such as recruitment strategies, barriers and facilitators, successes and unexpected occurrences. For example, in 1 case, patient representatives told the junior researcher primarily about problems of care in their own health‐care institute, which put the researcher in a kind of “therapist” role. In another case, a junior researcher explained that the patient felt “neglected.” One participant reported that the recruitment strategies were perceived as unexpectedly successful and the “right” patients were found easily by asking patients who had been interviewed in the previous stage of the research project.

*[name of junior researcher] is developing an intervention to improve after‐care for cancer patients. During the previous phase she did interviews. There were two interviewees that were very enthusiastic and they saw the bigger picture of cancer after‐care. These two patients were also invited by [name of junior researcher] to an expert meeting with health care professionals. She regrets that she did not ask them to review the questioning route for the focus group discussion that followed the expert meeting*. [minutes of project team meeting]

In one case, patient participants provided valuable information about the “social care network” in certain neighbourhoods that were involved in the intervention development. Finally, in another research project, aimed at evaluating an educational intervention for health‐care professionals, patients acted as simulated patients, with a predetermined topic but using their own story in the educational intervention. The junior researcher and the professionals perceived the coaching with real patients as educational, and the patients found it a rewarding experience. Junior researchers presented their PPI experiences, provided feedback to each other and shared practical tips. In some cases, they adjusted the design of PPI in their project during these coaching sessions. This resulted in 5 extensively described best practices for PPI, which were published in a separate booklet.

### 3.4 Senior researchers

After splitting up the group into 2 subgroups, the senior researchers reflected on their role and responsibilities as supervisors of juniors and managers of research in the fifth coaching session. Because the seniors had realized that they did not have an answer to some of the barriers identified by the junior researchers (Table 3), we invited 2 experts for a separate meeting, one leading investigator of another research programme focusing on “inclusion of vulnerable people” and 1 patient expert from Huis voor de Zorg with extensive research experience. There was, for instance, the issue around payment of patient representatives. From the literature, junior researchers learned that adequate acknowledgement of the contributions of patients should be considered at an early stage.^7^, ^35^, ^36^ When they tried to formulate the role description of patient representatives and were confronted with the question asked by the moderator to consider not only what they asked from patients but also what they could offer, it became clear that senior researchers had not been given guidance from their institution. Some senior researchers still felt insecure while some felt that PPI should be imperative for all research projects.

*[name of senior researcher] says that theoretically, it [PPI] is now much clearer to me, but in daily practice I still think it's hard work. ‘We're still not confident enough’. I want to know what they [patients] can do for us and who decides. [name of another senior researcher] replies that we as a research group must not do any research projects without patient involvement*. [minutes session]

The need for a clear vision and structure for PPI, including the available support and resources for PPI, prompted the seniors to initiate a white paper on the policy for PPI at their Research Centre. This white paper presents a vision on PPI and outlines an infrastructure for PPI that supports researchers in the practice of incorporating meaningful PPI in their research projects.

### 3.5 Concluding joint session of juniors, senior researchers and patients

The sixth and last coaching session was a joint session with representatives of all stakeholders, with the aim of encouraging direct dialogue between junior and senior researchers and patient representatives. The presence of most of the senior researchers demonstrated their commitment and function as role models to illustrate the importance of PPI in research. All juniors and 3 patient participants were present. Two patient participants could not attend.

The senior researchers had become aware of their role in enabling PPI and felt responsible for it. They started to work on establishing preconditions for connecting junior researchers, patients and research teams. Others maintained a “wait‐and‐see” approach, observed what happened and interfered and supported only when needed.

*[name of senior researcher] reflects on himself as a kind of meerkat. He understands his role as a meerkat guard who has a good vantage point to watch out for what is going on around him concerning PPI at the research centre. He supports PPI where needed but does not “actively” stimulate PPI forwards*. [session recording]

One senior referred to herself as a “newcomer” and preferred to follow the mainstream, as this role provided some safety.

The junior researchers mentioned that they had at first perceived PPI as very complex because it required a different responsibility in addition to the existing and partly simultaneous implementation activities. They had learned from the coaching programme that they were able to multitask with PPI, and their research became more patient‐friendly. Some described their role as “explorers,” focusing on discovering what PPI could offer and what added value it could bring to the research project. It became apparent that the juniors had broadened their scope as researchers by not only doing research, but also looking around and being curious about what happened in other research projects. They appreciated the interactive nature of the coaching programme.

*I learned a lot from the interactions with my colleagues. How they involved patients, what recruitment strategies they used, problems they encountered. It made me reflect on my own way of doing PPI, because of the interactive nature of the coaching*. [session recording]

One participant perceived PPI as a balancing act: adherence to robust methodological standards vs searching for ways to include new stakeholders in the research process.

All senior and junior researchers acknowledged that without this coaching programme, the level of PPI in their project would have remained at the level of a single consultation.

The patient representatives expressed appreciation of the option to choose a particular patient role in a research project. They also perceived the collaborative relationships as rewarding and fulfilling, being allowed to go through a process of growth as a research partner in the course of the research project to contribute to better patient care.

*(…) we as patient representatives learn about research. We need time to understand and comprehend research projects. To me, it's very worthwhile to be involved in research, as I feel I can contribute to better care and make a difference to those who are in need of care*. [session recording]

When asked to evaluate the coaching programme senior, they confirmed the importance of reminding researchers to coach patients in understanding the content of the research project, and of making senior and junior researchers aware of the need to create a supportive environment for patients to optimize their contributions.

Finally, the project team members felt pleased with the positive feedback and with the reported impact of the coaching programme on research projects, and with the individual, practical and structural changes that senior and junior researchers reported. Although not all juniors and seniors were able to attend all group meetings, there were no drop‐outs.

### 3.6 Key learnings

During project team meetings, we reflected regularly on the barriers and facilitators for creating a community of PPI practices and identified helpful conditions for developing a coaching programme for researchers and designing and implementing PPI in daily research practice (Table 4).

**Table 4:** Conditions for establishing a community of patient and public involvement (PPI) practice and developing a coaching programme

1. Full involvement of all stakeholders in the project team, including patient representatives, right from the start.
2. Commitment by the research centre's leadership.
3. A clear vision on PPI and the required strategies and resources for its implementation.
4. Constructing the road while walking on it: co‐creation of the coaching programme.
5. A tailor‐made approach that acknowledges the presence of contextual factors for PPI.
6. Benefits of combining group sessions and individual coaching to avoid drop‐outs.
7. Starting from the participants’ comfort zone.
8. Distinguishing PPI tasks and responsibilities of junior vs senior researchers.
9. Integrating the concept of the participation ladder in all programme components.
10. Involving patient representatives in the evaluation of the coaching programme.

Close collaboration with a regional patient organization proved to be of great value. The Huis voor de Zorg was a co‐initiator of our project and has contributed much to the formulation of role descriptions and the recruitment of patient research partners. The commitment of the research programme leader, principal investigators, a clear vision on PPI and the assurance that junior researchers were allowed to make mistakes, turned out to be important facilitators.

We followed the adage of constructing the road while walking on it: we recommended that senior and junior researchers should start on a small scale and discuss opportunities and concerns with patients along the way. Coaching proved to be a feasible approach to building the programme and incorporating PPI skills training, building a supportive attitude towards PPI and imparting knowledge of PPI concepts and methods. For this reason, we did not pressurize senior and junior researchers to do something they were not familiar with. We started from the comfort zone of researchers and then, by means of competence‐based learning, gradually increased their expectations by exploring the boundaries of their comfort zone. This endeavour required a balanced approach that taught them the evidence‐based concepts of PPI while at the same time respected the contextual factors that are important in conducting PPI. We believe that focusing on the context and particularities of the research projects avoided drop‐out.

We also learned the importance of differentiating between the tasks and responsibilities of senior and junior researchers. As introduced in the FIRST model^5^ and elucidated in a follow‐up study,^7^ supporting PPI is not the same as facilitating. Providing this coaching programme is an example of facilitating PPI, meaning that the institute's leadership acknowledged the need for PPI coaching for researchers, and assumed responsibility for providing the resources to make this happen.

## 4 DISCUSSION

Nowadays, researchers are expected to include the voices of patients in their projects, but acquiring the necessary competence to engage patients in a meaningful way is seldom integrated in their curriculum. In this article, we have described an interactive and iterative approach to PPI coaching development using action research and at the same time establishing a community of PPI practice. This coaching programme was developed in close collaboration with all stakeholders and researchers motivated to implement meaningful PPI. The enthusiasm generated in the first coaching session motivated senior researchers to start up a second and third coaching programme. This bottom‐up coaching approach may help other managing researchers to design and implement customized PPI coaching at their own departments and research centres.

A strength of our approach is the reversed role of a patient expert as educator and coach of researchers. This is a novelty because we have become used to researchers educating patients how to contribute to the research process. Inviting a patient representative with extensive practical as well as academic expertise in PPI to the project team ensured a strong patient focus throughout the development process. Another strength of this study is its supportive outcomes, such as the institute's white paper on PPI, a toolkit for PPI (including the participation game, participation matrix and guide for coaches) and a policy document on funding and reimbursement. These materials represent the start and gradual development of a community of PPI practice. Our coaching approach confirms the benefits reported in the literature, such as an increased quality of research due to a better understanding of the process and a reduced need for frequent and intensive supervision. Researchers gain confidence and become more efficient in collaborating with patients.^37^

This study also has a few limitations, which can inform the future research agenda. First, we have not yet systematically evaluated the effectiveness of the coaching programme in terms of improved competences of the researchers, the sustainability of cultural change and the programme's cost‐effectiveness. Despite the positive outcomes reported, the willingness and ability of future researchers to engage patients in this role are not self‐evident. It still requires an extra effort from senior researchers to establish a stimulating environment for PPI and to enable junior researchers to acquire the competences to ensure the sustainability of the PPI outcomes. These efforts go beyond the level of the individual researcher and include changes at the level of the research team and the culture and organization of the institute.^3^ A community of PPI practice can only flourish when a culture arises in which the added value of PPI is no longer contested, but in which researchers feel responsible and committed to sharing best practices and providing advice and support to colleagues. The next step is to validate the coaching programme or the approach of establishing a community of practice through reflective action research by exploring its transferability to other research contexts. This could provide more information about the value of simultaneously coaching researchers and patient participants, as suggested in other studies.^17^ It should also reveal the potential pros and cons of coaching junior and senior researchers separately. We have observed that junior researchers, like patient representatives, need a safe environment to report and discuss personal experiences and challenges of PPI. Bringing patients and researchers together at an earlier stage to reflect on the collaboration might be possible and advantageous, but changes should be carefully considered.

Although patient experts were involved in the project team and patient participants were invited for the final session, a more active involvement of patients in the development and implementation of the coaching programme could have made a difference. There are studies that emphasized the pivotal role of direct dialogues between patients and researchers in the process of co‐creation. Several successful case studies of developing shared research agendas have been published.^38^, ^39^ One can even argue that the active involvement of patients should be mandatory when developing a community of PPI practice in this field.

We conclude that a stepwise approach, based on action research, coaching and dialogue between peers, empowers senior and junior researchers to acquire concepts and tools to engage patients in a meaningful way. We recommended distinguishing between the roles and responsibilities of senior and of junior researchers.

## CONFLICT OF INTEREST

There was no financial support or other benefits from commercial sources for the work reported on in the manuscript, or any other financial interests that any of the authors may have, which could create a potential conflict of interest or the appearance of a conflict of interest with regard to the work.

## Note

1 The term patients is used as synonym of clients, end‐users and health consumers. We also include carers, family members or health professionals who represent patients unable to speak for themselves.
